# KEAP1 Cancer Mutants: A Large-Scale Molecular Dynamics Study of Protein Stability

**DOI:** 10.3390/ijms22105408

**Published:** 2021-05-20

**Authors:** Carter J. Wilson, Megan Chang, Mikko Karttunen, Wing-Yiu Choy

**Affiliations:** 1 Department of Biochemistry, The University of Western Ontario, 1151 Richmond Street, London, ON N6A 5C1, Canada; cwils256@uwo.ca (C.J.W.); mchang96@uwo.ca (M.C.); 2 Department of Applied Mathematics, The University of Western Ontario, 1151 Richmond Street, London, ON N6A 5B7, Canada; 3 Department of Chemistry, The University of Western Ontario, 1151 Richmond Street, London, ON N6A 3K7, Canada; 4 Centre for Advanced Materials and Biomaterials Research, The University of Western Ontario, 1151 Richmond Street, London, ON N6A 5B7, Canada

**Keywords:** KEAP1, NRF2, cancer, protein stability, biophysics, bioinformatics, molecular dynamics, simulation

## Abstract

We have performed 280 μs of unbiased molecular dynamics (MD) simulations to investigate the effects of 12 different cancer mutations on Kelch-like ECH-associated protein 1 (KEAP1) (G333C, G350S, G364C, G379D, R413L, R415G, A427V, G430C, R470C, R470H, R470S and G476R), one of the frequently mutated proteins in lung cancer. The aim was to provide structural insight into the effects of these mutants, including a new class of ANCHOR (additionally NRF2-complexed hypomorph) mutant variants. Our work provides additional insight into the structural dynamics of mutants that could not be analyzed experimentally, painting a more complete picture of their mutagenic effects. Notably, blade-wise analysis of the Kelch domain points to stability as a possible target of cancer in KEAP1. Interestingly, structural analysis of the R470C ANCHOR mutant, the most prevalent missense mutation in KEAP1, revealed no significant change in structural stability or NRF2 binding site dynamics, possibly indicating an covalent modification as this mutant’s mode of action.

## 1. Introduction

One of the major open problems in computational structural biology is predicting the three-dimensional folded structure of a protein given its primary amino acid sequence. This has remained practically unsolved for over 50 years [1], but 2020 ushered in a major change: DeepMind’s AlphaFold 2, which utilizes a deep learning system, spectacularly outperformed all other groups at the 14th Critical Assessment of protein Structure Prediction (CASP) [2,3]. DeepMind’s paradigm-shifting breakthrough at CASP allowed them to predict the three-dimensional structure of a protein given its primary amino acid sequence with accuracy comparable to experimental methods. Herein, we consider a similarly intriguing sub-problem, predicting the change in the three-dimensional structure of a protein under various amino acid substitutions, given its initial folded structure.

It has been well documented that many diseases, including cystic fibrosis [4], Parkinson’s [5], Rett syndrome [6] and some cancers [7], arise due to a single or only a handful of amino acid substitutions in key proteins. These mutations tend to either disrupt the delicate intramolecular scaffolding of a protein, destabilizing the folded state [8,9] or impair the intermolecular interactions between a protein and its binding partners, often giving rise to aberrant gene product production [10,11]. It is, therefore, no surprise that in the advent of personalized medicine, the characterization of a patient’s unique proteome concerning the stability and target binding of key proteins is highly desirable for the design of novel therapeutics and treatment protocols [12,13,14,15,16,17,18].

The development of in silico methods has primarily focused on either predicting the effect of mutations on stability [19] or binding affinity [20]. With respect to stability, the methods employed can be broadly classified as either sequence- or structure-based. Sequence-based methods consider only the primary amino acid sequence. Historically, these have employed sequence alignments and homology searches [21,22] but have more recently employed machine-learning (ML) approaches [23,24,25,26]. Structure-based methods consider the known three-dimensional structure of a protein and utilize potential-energy-based approaches [27,28,29], structural modeling and sampling [30,31], normal mode analysis [32], and ML techniques [33,34,35,36,37,38] to predict the impacts of mutations. While structure-based methods tend to perform better than sequence-based methods, meta-analyses have demonstrated the failure of these methods to accurately characterize mutations of buried amino acids [39], and adequately control for the bias introduced by the training sets, which can often lack key thermodynamic parameters [15,19,40,41,42,43]. As a result, even the best predictive methods have historically shown only about 60% accuracy [40,44,45,46].

Molecular dynamics (MD)-based methods have been increasingly successful in predicting the effects of point mutations on protein stability and, unlike static in silico methods, can provide detailed atomistic information concerning wild-type and mutant protein dynamics [47,48,49,50,51,52,53,54,55], mutagenic changes in stability [56,57,58,59] and the effects of mutation on target binding [60,61,62,63,64]. These insights may be difficult to obtain by experimental techniques and are often beyond the reach of conventional in silico methods. As a result of increased computational power, MD simulations have begun to reach experimentally relevant time scales at the millisecond level [65].

Kelch-like ECH-associated protein 1 (KEAP1) is an oxidative stress sensor, functioning as an adaptor for the Cullin-3 (CUL3) ubiquitin ligase, which regulates the activity of nuclear factor erythroid 2-related factor 2 (NRF2), the master regulator of cytoprotective gene expression [66]. Under resting conditions, KEAP1 dimerizes and the Kelch domain of each protein monomer binds to one of the two motifs (ETGE or DLG) in NRF2’s Neh2 domain. This sequestering of NRF2 facilitates its ubiquitination and subsequent proteolysis, therefore repressing cytoprotective gene expression [67]. Under oxidative stress, key solvent-accessible cysteine residues in KEAP1 are post-translationally modified, resulting in the release of NRF2 by KEAP1, increased translocation of NRF2 to the nucleus and upregulation of cytoprotective genes [67]. Studies have shown that mutations at the KEAP1-NRF2 interface are prevalent in cancer, and it is believed that some of the genes regulated by NRF2 confer a chemoprotective phenotype to cancer cells [68].

**Figure 1:**
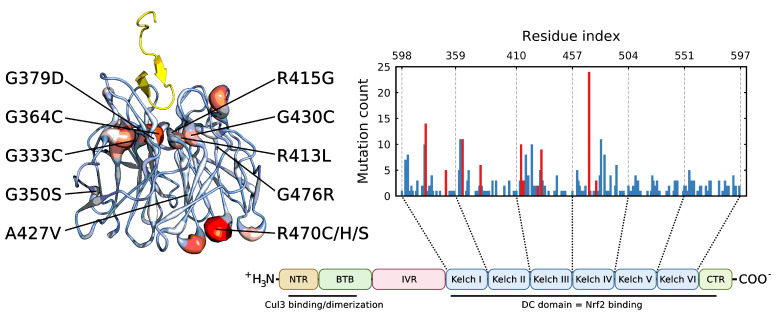
Mutant locations and frequency in the Kelch domain. β-propeller Kelch domain of KEAP1 bound to the 16-mer peptide encoding the ETGE motif from the Neh2 domain of NRF2 (yellow). The putty structure describes the mutational frequency at the given position (red/large = high frequency, blue/small = low frequency). The 12 cancer mutants are listed and indicated. Barplot shows the COSMIC [69] mutation frequency and corresponding location within the various blades (I-VI) of the β-propeller structure. Red bars indicate the mutants studied in this work. Residues that roughly separate the blades are indicated; note that blade I is a “junction blade” containing residues from the N- and C-termini (325–358 and 598–609). The Cullin-3 and NRF2 binding regions are also depicted.

Intriguingly, not all mutations in KEAP1 reside at this interface and the missense mutation most frequently documented in KEAP1 in the COSMIC [69] cancer database is R470C, which is curiously located distantly from the NRF2 binding site (Figure 1). A handful of similar mutants have been identified and coined “additionally NRF2-complexed hypomorphs” (ANCHOR mutants) and appear to confer an increased affinity to KEAP1 for NRF2 [70]. A previous study postulated that these mutations have an allosteric effect via fluctuations that propagate through the Kelch domain and alter the binding site [71]. This has yet to be verified or in silico.

In this work, we performed over 280 μs of MD simulations to characterize 12 key KEAP1 mutations that were chosen as a representative sample of the total mutations present in the COSMIC database for the Kelch domain of KEAP1, and for which some previous experimental binding and structural data were available [70,71,72]. For nine of these mutants (G333C, G350S, G364C, G379D, R413L, G430C, R415G, A427V and G476R) nuclear magnetic resonance (NMR) spectroscopy and isothermal titration calorimetry (ITC) were used in an attempt to elucidate their effects on stability and target binding. Notably, five of the nine mutants (G333C, G379D, R413L, G430C and G476R) were insoluble and could not be characterized, leaving a significant knowledge gap [72].

Our results demonstrate that MD is capable of predicting the effects of mutations on protein stability and can provide structural insights that are often difficult to gain from static predictive methods. Specifically, we found that blades I, II and III in the Kelch β-propeller structure appear to be the least stable, which, taken together with their observed mutational frequency, points to stability as a possible target of cancer in the Kelch domain of KEAP1. On the other hand, our structural and binding site analysis revealed no significant change in structural fluctuations resulting from the R470C ANCHOR mutation, suggesting that another mechanism, such as an covalent modification of the cysteine, may be this mutant’s mode of action.

## 2. Methodology

### 2.1. System Setup

The starting protein structure was prepared from the Neh2-Kelch crystal structure (PDB: 2FLU) [73] downloaded from the Protein Data Bank [74]. The 16-mer ETGE peptide was removed from the complex, leaving only the Kelch domain to be simulated. MODELLER [75] was used to rebuild missing atoms, and PyMOL [76] was used to perform residue substitution producing 13 unique structures (12 mutants and wild type). MD simulations were performed using GROMACS 2018.7 [77] with the CHARMM36m forcefield [78] under periodic boundary conditions. The protein was centered in a rhombic dodecahedral box such that all atoms were positioned at least 2.0 nm from the box edge. The box was solvated using TIP3P explicit water [79] and K${}^{+}$ ions (6–8 depending on the system) were added to maintain overall charge neutrality. The protonation states of all ionizable residues were chosen on the basis of their most probable state at pH 7, and all simulations were conducted with the amino and carboxyl terminal ends of the protein left uncapped (NH3${}^{+}$ and COO${}^{-}$, respectively).

### 2.2. Simulation Protocol

Each system contained approximately ∼57,000 atoms. Prior to equilibration, we used the steepest descents algorithm to minimize the energy of each system. A single 100 ps equilibration was run in the NPT ensemble at 310 K and 1 bar following energy minimization to ensure system stability. During production runs, a constant temperature of 310 K was maintained using the Parrinello–Donadio–Bussi velocity rescaling method [80] with a coupling time of 1 ps; this approach has previously been shown to perform well for similar biomolecular simulations [81,82]. The 310 K temperature was chosen to match physiological conditions. A Parrinello–Rahman barostat [83] with a coupling time constant of 5 ps was used to maintain the pressure at 1 bar. The Particle-mesh Ewald (PME) method [84] with a Fourier spacing of 0.12 nm, and a real-space cut-off of 1.0 nm was used to calculate the long-range electrostatic interactions. A 1.2 nm cut-off was used for the Lennard-Jones interactions. Hydrogen bond lengths were constrained using the LINear Constraint Solver (P-LINCS) [85]. The simulation time step was 2 fs. All mutant structures were simulated for 10 μs with one replicate (2 runs total), while the wild type was simulated for 10 μs with three replicates (4 runs total). The total simulation run time was 280 μs.

### 2.3. Analyses

The Kelch domain is a β-propeller containing six blades connected by linkers (Figure 1). For analysis purposes, these blades are defined accordingly: blade I [(325–358, 598–609)], blade II [(359–409)], blade III (410–456), blade IV (457–503), blade V (504–550) and blade VI (551–597). Note that blade I is a “junction blade” containing residues from the N- and C-termini of the protein. To discern the global and local structural fluctuations of the protein, the root mean square deviation (RMSD), moving root mean square deviation (mRMSD) and root mean square fluctuation (RMSF) were calculated for all backbone atoms following a least squares fit to the backbone of the reference structure. Given the structure of the Kelch domain, a natural parameter choice is the anti-parallel β-sheet order parameter (S${}_{a\beta }$). S${}_{a\beta }$ measures the RMSD between all 6 residue segments in a protein and an idealized anti-parallel β-sheet. The original formulation was given by Pietrucci and Laio [86],

(1) $$S_{a\beta }=\sum_{i}^{N}\frac{1-{\left(\frac{r_{i}-d_{0}}{r_{0}}\right)}^{n}}{1-{\left(\frac{r_{i}-d_{0}}{r_{0}}\right)}^{m}},$$

where $r_{i}-d_{0}$ is the backbone RMSD (in nm) of a given 6-residue fragment *i* from an ideal β-sheet, *N* is the number of overlapping six-residue segments in the protein, and $r_{0}=0.8$, n=8, and m=12 are parameters defined in the original work [86]. A completely anti-parallel, β-sheeted structure will have an S${}_{a\beta }$ value near *N*, while a completely disordered conformation will have an S${}_{a\beta }$ value near zero. We used the PLUMED ANTIBETARMSD collective variable [87,88] to perform the relevant calculations post hoc. Block averaging was used to compute both the mean and standard deviation over the sampled trajectories. Dihedral angle and hydrogen bonding analyses were performed using the MDAnalysis package [89,90]. pKa analysis was performed using the DelPhiPKa webserver [91,92]. Default settings were used for all non-MD predictor methods. Three-dimensional protein structure figures were generated using PyMOL [76].

## 3. Results

### 3.1. Mutations Have Differential Effects on Kelch’s β-Sheets

**Figure 2:**
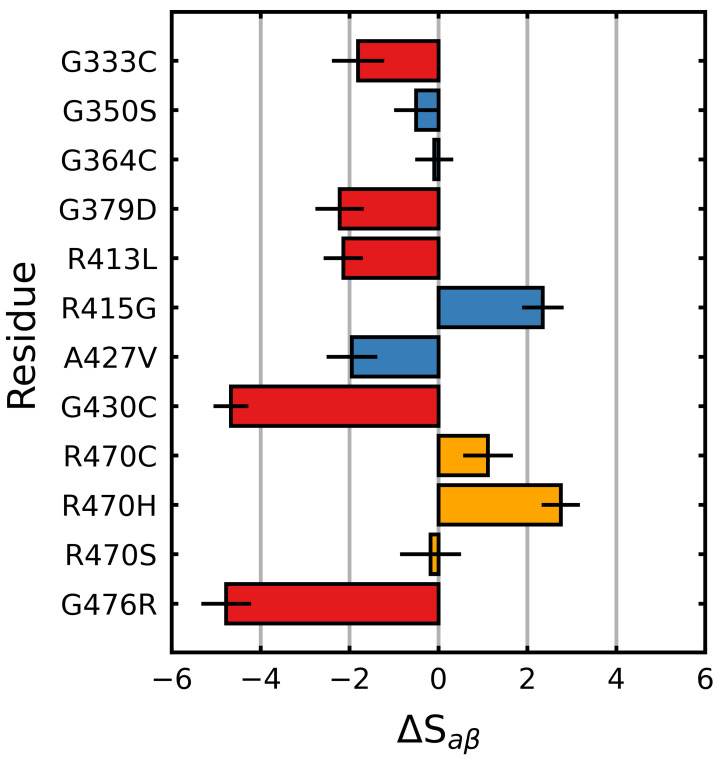
Mutagenic effect on S${}_{a\beta }$ of Kelch. The average change in S${}_{a\beta }$ (Equation (1)), relative to the wild type was calculated and plotted along with the standard deviation. The colors of the bars denote the documented effect of the given mutation [70,71,72]: red (destabilizing), blue (neutral/stabilizing) and orange (unknown or neutral/stabilizing). Sampling was performed over the final 5 μs.

S${}_{a\beta }$ (Equation (1)) was calculated over the final 5 μs of each trajectory in order to characterize the effects of the mutations on the Kelch β-propeller, a collection of anti-parallel β-blades. We found that the experimentally characterized destabilizing mutations (G333C, G379D, G430C, R413L and G476R) tended to decrease S${}_{a\beta }$ relative to the wild type, while the neutral/stabilizing mutations (G350S, G364C, R415G, A427V and R470C/H/S) tended to increase it or had little effect. All the destabilizing mutations resulted in a two or more point decrease in S${}_{a\beta }$ ($\Delta S_{a\beta }<-2$), while the G430C and G476R mutations resulted in decreases of more than four points (Figure 2).

With respect to the neutral/stabilizing mutations, A427V and G350S resulted in moderate decreases in $S_{a\beta }$ ($\Delta S_{a\beta }\geq -2$), while G364C and R415G resulted in no change and an increase, respectively. Notably, the ANCHOR mutation R470C and its variant R470H resulted in increases in $S_{a\beta }$.

**Figure 3:**
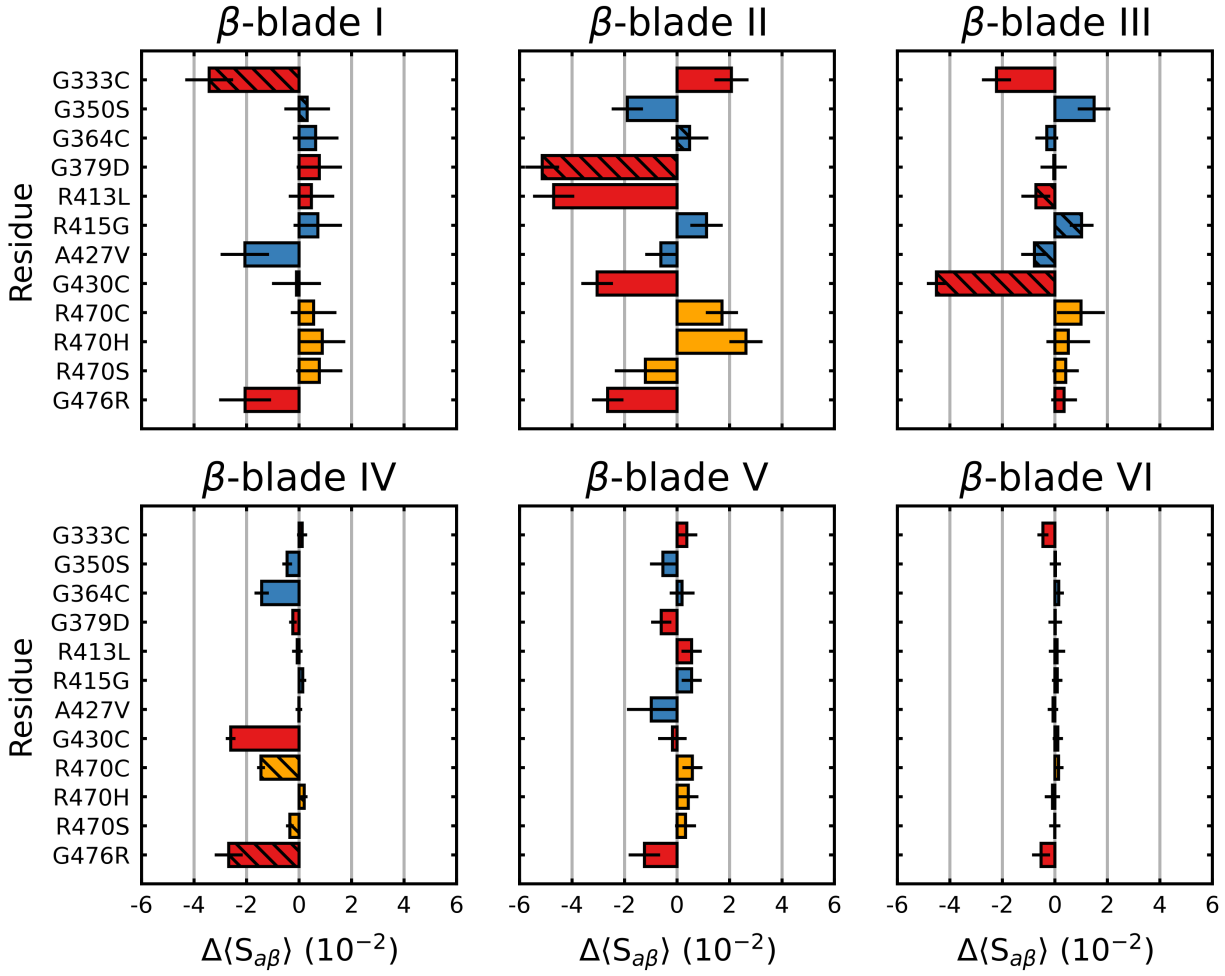
Mutagenic effect on the blade-wise S${}_{a\beta }$ of Kelch. The average change in S${}_{a\beta }$ (Equation (1)) (per residue), relative to the wild type was calculated and plotted along with the standard deviation for each β-blade. The colors denote the documented effect of the given mutation [70,71,72]: red (destabilizing), blue (neutral/stabilizing) and orange (unknown or neutral/stabilizing). Hatched bars indicate that the mutation is within that particular blade. Sampling was performed over the final 5 μs.

**Figure 4:**
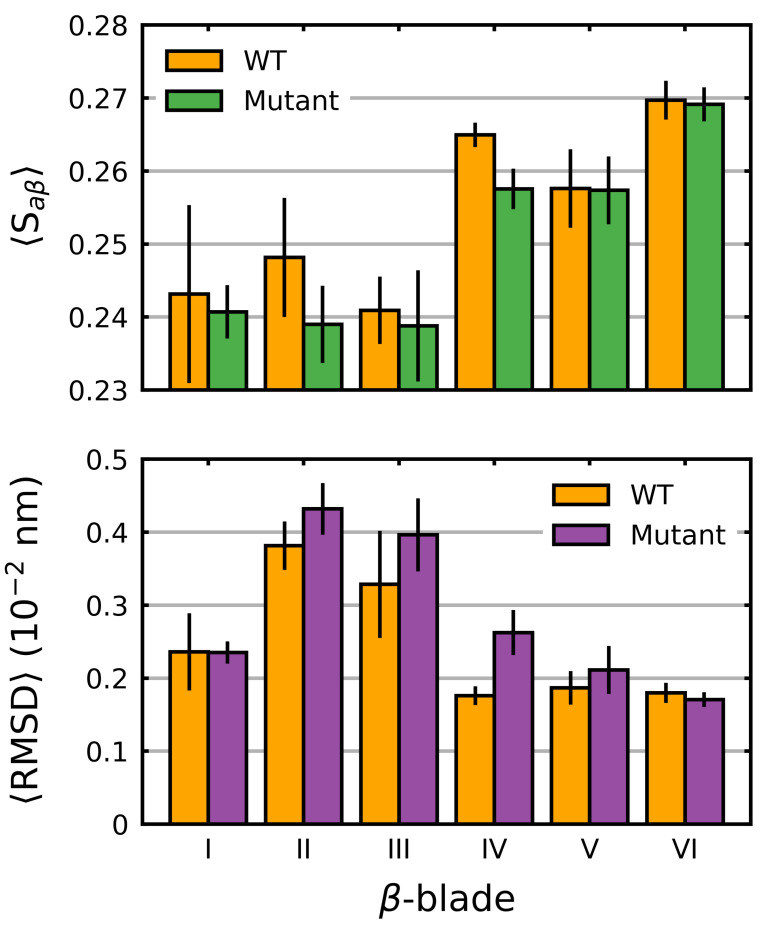
Average blade-wise S${}_{a\beta }$ and RMSD in the Kelch domain. The average S${}_{a\beta }$ (Equation (1)) (per residue) and RMSD (per residue) was calculated and plotted along with the standard deviation for each β-blade in the wild-type Kelch. The average over all the mutants (both stabilizing and destabilizing) was also calculated and plotted. Sampling was performed over the final 5 μs.

We also considered the individual blades with respect to $S_{a\beta }$. In these cases, a per-residue $S_{a\beta }$ (denoted herein as 〈S${}_{a\beta }\rangle$) was used to account for the small differences in the individual blade lengths within the Kelch propeller. Unsurprisingly, the blade-wise effects of the mutations tended to correlate with their location (Figure 3). Those blades where the destabilizing mutations were directly or closely situated, G333C (blade I), G379D (blade II), R413L (blade II-III linker loop) and G430C (blade III), showed the largest respective decreases. Interestingly, G476R located in β-blade IV, resulted in decreases in all but one of the six blades. Collectively blades I, II and III showed relatively larger increases compared to blades IV, V and VI irrespective of mutation. This was also true of the wild-type structure, which showed a blade-dependent $S_{a\beta }$, where the first three blades of Kelch had moderately lower 〈S${}_{a\beta }\rangle$ values than the final three (Figure 4).

### 3.2. Stabilizing and Destabilizing Mutations Both Result in Global Structural Deformation

**Figure 5:**
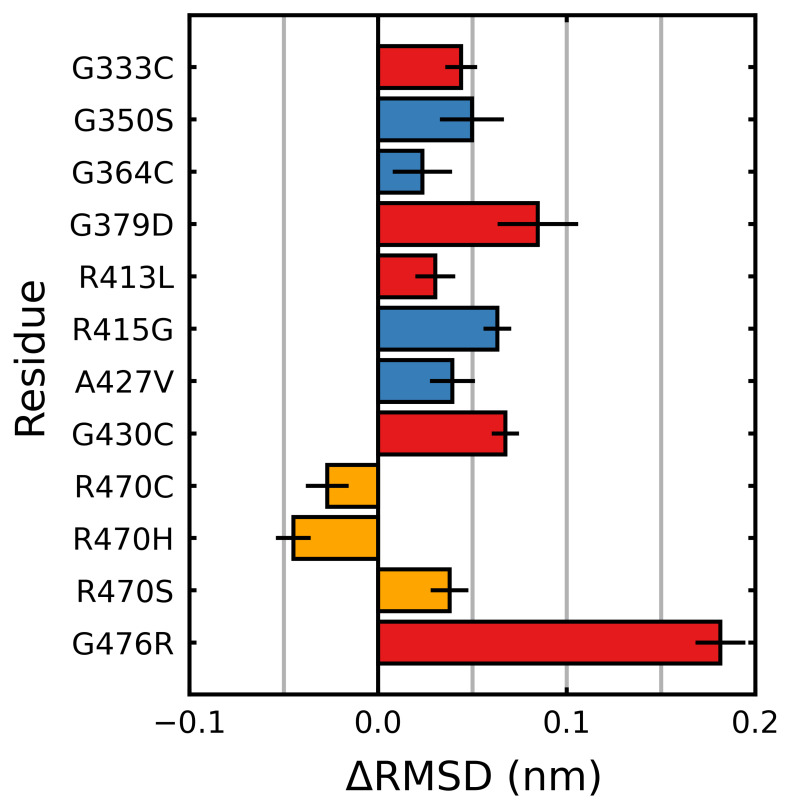
Mutagenic effect on RMSD of Kelch. The average change, relative to the wild type, of the RMSD was calculated and plotted along with the standard deviation. The colors denote the documented effect of the given mutation [70,71,72]: red (destabilizing), blue (neutral/stabilizing) and orange (unknown or neutral/stabilizing). Sampling was performed over the final 5 μs.

**Figure 6:**
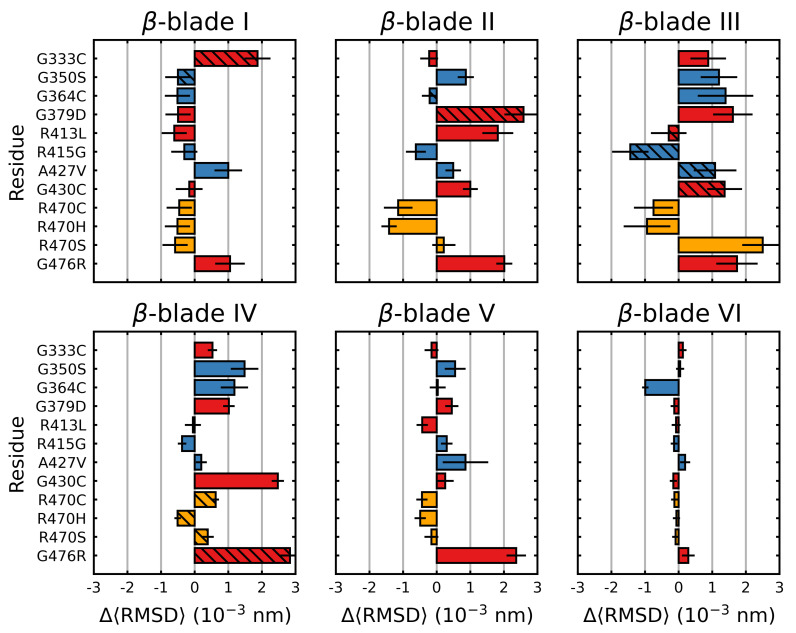
Mutagenic effect on the blade-wise RMSD of Kelch. The average change of the RMSD (per residue), relative to the wild type was calculated and plotted along with the standard deviation for each β-blade. The colors denote the documented effect of the given mutation [70,71,72]: red (destabilizing), blue (neutral/stabilizing) and orange (unknown or neutral/stabilizing). Hatched bars indicate the mutation is within that particular blade. Sampling was performed over the final 5 μs.

The root mean square deviation (RMSD) was calculated over the final 5μs of each trajectory. The RMSD is a measure of the average deviation of all the backbone atoms from their initial positions. A higher relative value indicates increased structural deviation and deformation, while a lower relative value indicates reduced deviation. For all but three mutations, larger deviations than the wild type were observed, with mutations G476R and G379D showing the greatest deviations (Figure 5). R470C and R470H were the only mutations that showed less conformational fluctuation than the wild type. We also considered the individual blades with respect to the RMSD. In these cases, a per-residue RMSD (denoted herein as 〈RMSD〉) was used to account for the small differences in the individual blade lengths within the Kelch propeller. Unlike 〈S${}_{a\beta }\rangle$, the destabilizing and neutral/stabilizing blade-wise effects of the mutations were less correlated with their location and documented effect (destabilizing: G333C, G379D, G430C, R413L and G476R; stabilizing/netural: G350S, G364C, R415G, A427V and R470C/H/S) [70,71,72] (Figure 6).

While overall the destabilizing mutations tended to increase deviations, particularly in those blades where they are directly or closely situated, some notable behavior was observed. In β-blades III and IV, three neutral/stabilizing mutations G350S, G364C and A427V resulted in increases in the RMSD. We also note that the G476R mutation in β-blade IV resulted in notable deviations in all six blades. Similar to 〈S${}_{a\beta }\rangle$, irrespective of mutation, blades II and III of Kelch had higher 〈RMSD〉 values than the others (Figure 4). The RMSD, mRMSD and radius of gyration were also computed in a time-dependent fashion (Figures S1–S4).

### 3.3. Destabilizing Mutations Shift the Backbone Dihedrals of Kelch

**Figure 7:**
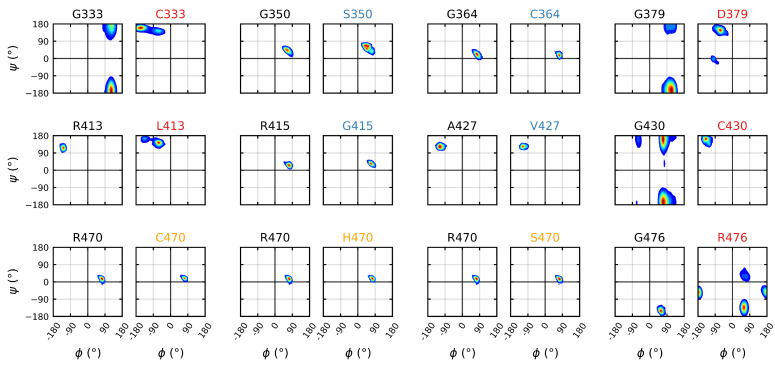
Mutagenic effect on the backbone dihedrals of the Kelch domain. The Ramachandran plots of the dihedrals for all mutations. The left plot depicts the wild-type dihedral space and the right plot the mutant dihedral space. The colors of the plot titles denotes the documented effect of the given mutation [70,71,72]: red (destabilizing), blue (neutral/stabilizing) and orange (unknown or neutral/stabilizing). A bivariate kernel density estimate [93,94] was used for plotting with a blue through red frequency map. Sampling was performed over the final 5 μs of trajectory.

The backbone torsion angles were calculated over the final 5μs of each trajectory in order to elucidate the effects, or lack thereof, of the various mutations on the wild type dihedral structure. The analysis indicated that while none of the neutral/stabilizing mutations had a significant effect on the dihedral sampling space, all of the destabilizing mutations resulted in significant shifts (Figure 7); the sampling spaces of G333C, G379D and G430C all shifted from a glycine allowable region (right hand side of the plot) to a β-sheet region under mutation. Unlike the other structural glycines, the sampling of the G476R mutation remained in the glycine allowable region; however, a much larger space was sampled therein. The R413L mutation resulted in the smallest shift among the destabilizing mutations, continuing to sample within the β-sheet region.

### 3.4. Loss of Hydrogen Bonds Due to Destabilizing Mutations

All potential hydrogen bonds (main-chain/main-chain, main-chain/side-chain and side-chain/side-chain) involving the mutated residues and their corresponding proportional lifetimes relative to the wild type were calculated over the final 5μs of each trajectory in order to elucidate their effects on the the hydrogen bond network of the Kelch domain. Exact lifetimes and hydrogen bonding partners are provided in Table S1.

**Figure 8:**
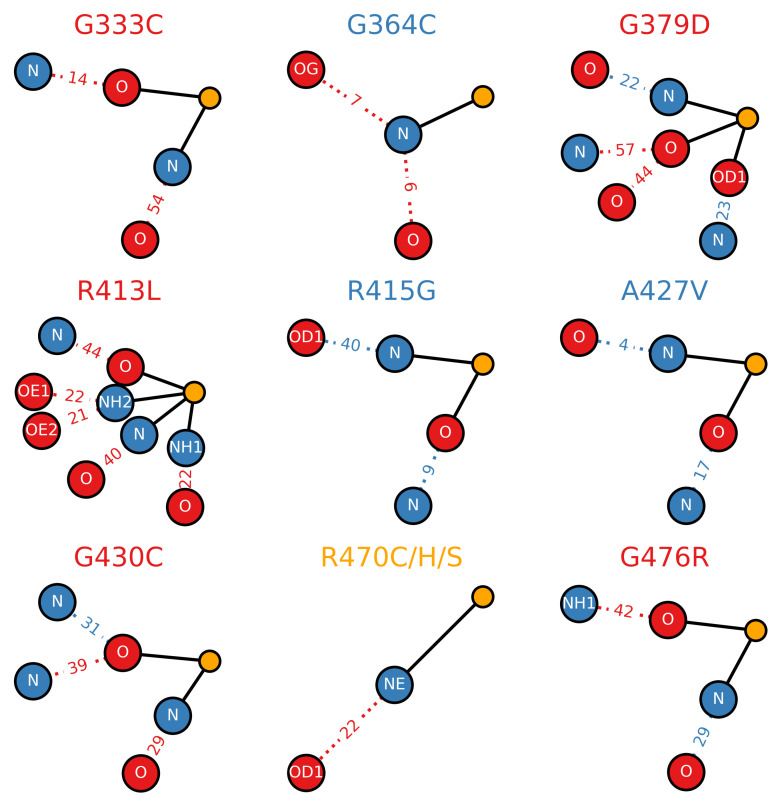
Mutagenic effect on the hydrogen bonding network of the Kelch domain. Wild type or mutant hydrogen bonds that existed for ≥20% of the sampled trajectory are considered in the calculation. Heavy atoms involved in bonding are connected via solid lines to a central orange “atom” while corresponding bonding partners are connected via dotted lines. Relative hydrogen bond existence times as a percent of the total trajectory (increase relative to wild type: blue, decrease relative to wild type: red) are indicated along each dotted line (i.e., |32.3% (mutant lifetime)–38.5% (wild type lifetime)|=|−6.2|≈6 decrease). The colors of the plot titles denote the documented effect of the given mutation [70,71,72]: red (destabilizing), blue (neutral/stabilizing) and orange (unknown or neutral/stabilizing). Sampling was performed over the final 5 μs of trajectory.

The destabilizing mutants R413L and G333C both resulted in significant drops in the hydrogen bond lifetime relative to the wild type (Figure 8). Conversely, the stabilizing mutants R415G and A427V both resulted in increases in the lifetimes. The ANCHOR mutation R470C resulted in a decrease in the hydrogen bond lifetime with D422.

**Figure 9:**
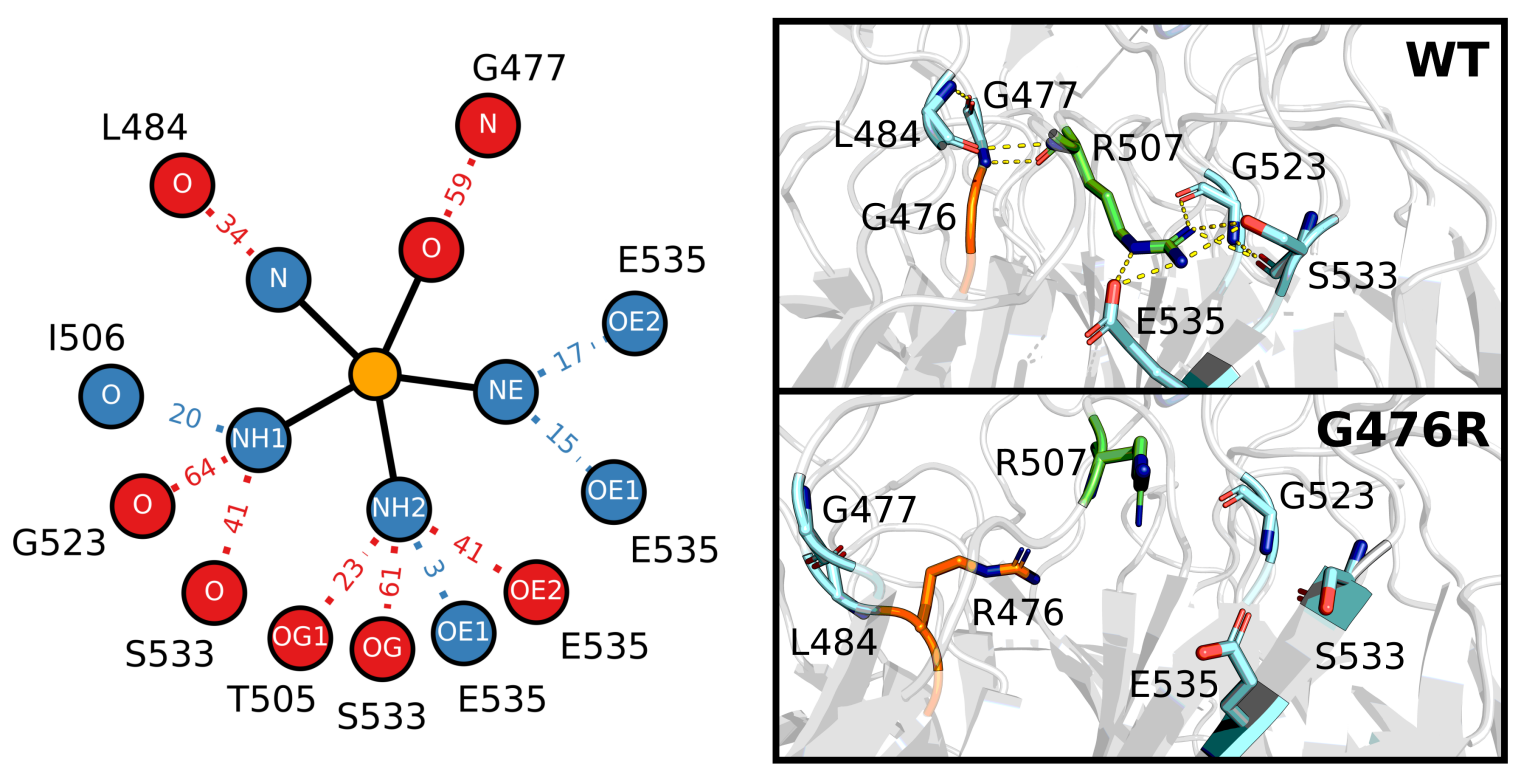
Effect of G476R mutant on the hydrogen bonding of R507. The left panel shows the hydrogen bond network of R507. Wild-type and G476R mutant hydrogen bonds that existed for ≥10% of the sampled trajectory are considered in the calculation. Heavy atoms of R507 involved in bonding are connected via solid lines to a central orange “atom”, while corresponding bonding partners are connected via dotted lines. Relative hydrogen bond existence times as a percentage of the total trajectory (increase relative to wild type: blue, decrease relative to wild type: red) are indicated along each dotted line (i.e., |32.3% (mutant lifetime)–38.5% (wild type lifetime)|=|−6.2|≈6 decrease). Sampling was performed over the final 5 μs of trajectory. The right panel shows the three-dimensional position of key hydrogen bonding partners of R507. The top structure is the PDB of the Kelch domain (ID: 2FLU [73]), while the bottom structure is taken from the final frame of one of the G476R trajectories. Orange atoms belong to the G476 or R476 residue, green atoms to R507 and cyan atoms to the hydrogen bonding partners. Predicted hydrogen bonds are depicted in the top structure with yellow dashes.

Not all the mutations directly affected the hydrogen bonding network of Kelch. The notable increases in the RMSD, and decreases in S${}_{a\beta }$, as well as the unique backbone dihedral pattern of G476R (Figure 7), led us to take a closer look at this buried amino acid. We identified a nearby arginine (Figure 9), R507, that forms hydrogen bonds with G477, L484, G523 and S533, all of which were significantly disrupted or completely lost as a result of the G476R mutation. Exact lifetimes and hydrogen bonding partners are provided in Table S2. Analysis of the residue-wise contact map revealed similar trends to those observed in the hydrogen bonding (Figure S5).

### 3.5. Limited Fluctuations at Both the ANCHOR-Mutant Mutation Site and NRF2 Binding Site

**Figure 10:**
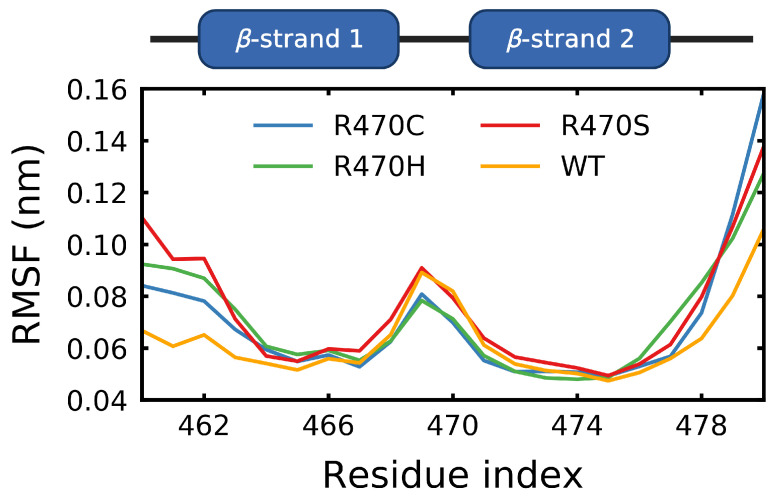
Fluctuations at the ANCHOR mutation site. Sampling was performed every 10 ns over the final 5 μs of trajectory. The root mean square fluctuations of the wild type and the ANCHOR mutant (R470C) and variants (R470H/R470S) were calculated at the mutation site. The mutation lies in the β-turn region connecting two strands in blade IV of the Kelch domain. Sampling was performed over the final 5 μs of trajectory.

**Figure 11:**
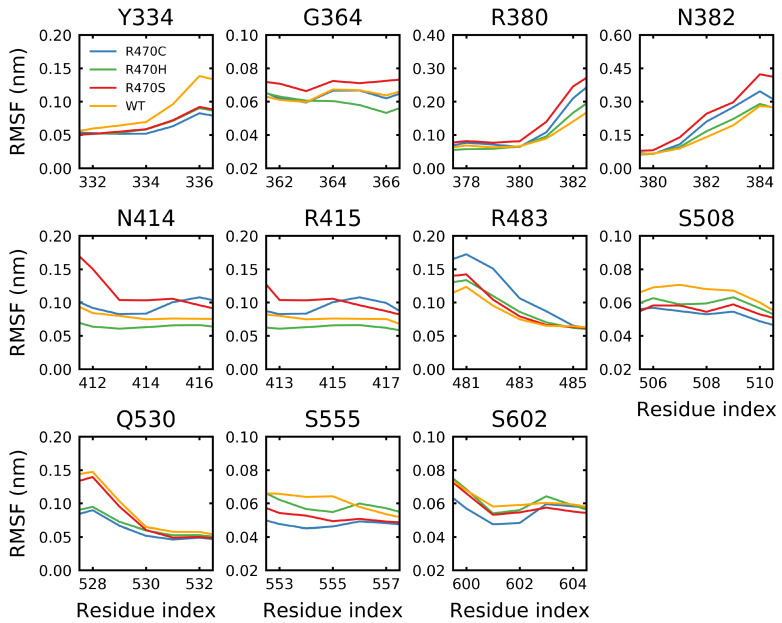
Effect of the ANCHOR mutations on binding site fluctuations. The root mean square fluctuations of the wild type and the ANCHOR mutant (R470C) and variants (R470H/R470S) was calculated at the residues involved in binding the ETGE (PDB: 2FLU [73]) or DLG (PDB: 3WN7 [96]) peptides as determined by PISA [95]. Sampling was performed over the final 5 μs of trajectory.

The root mean square fluctuation (RMSF) was calculated over the final 5μs of the R470C, R470H, R470S and wild-type trajectories. The RMSF is a measure of the average geometric deviation of a residue from its initial position. An RMSF near zero indicates that a residue spends most of the time constrained to its initial position. Unstructured loop and coil regions tend to have higher RMSF values, while structured helical and β-sheet regions tend to have lower values. The RMSF at and around the R470C, R470H and R470S mutation sites resulted in minimal changes from the wild type; however, the fluctuations at R470C and R470H were the lowest of the mutations considered (Figure 10). Notably, all of the R470 mutations resulted in significantly higher fluctuations at residues flanking the mutation site (i.e., <463 and >477) compared to the wild type. The ANCHOR mutants are believed to increase binding affinity of KEAP1 for NRF2 via backbone perturbations that alter the binding site [70]. Residues identified by PISA (Proteins, Interfaces, Structures and Assemblies) [95] to be involved in hydrogen-bonding with the Neh2 domain of NRF2 (Y334, G364, R380, N382, N414, R415, R483, S508, Q530, S555 and S602) were examined. While only minor effects due to the mutations were observed, the R470H and R470C mutations tended to show the lowest fluctuations at binding site residues (Figure 11). For the Y334, S508 and S555 residues, reduced fluctuations were observed for all the mutations compared to the wild type. Notably, the R470C mutation resulted in the highest fluctuation at R483, which is the closest binding site residue in the protein sequence.

## 4. Discussion

### 4.1. Structural Mutation Prediction

**Figure 12:**
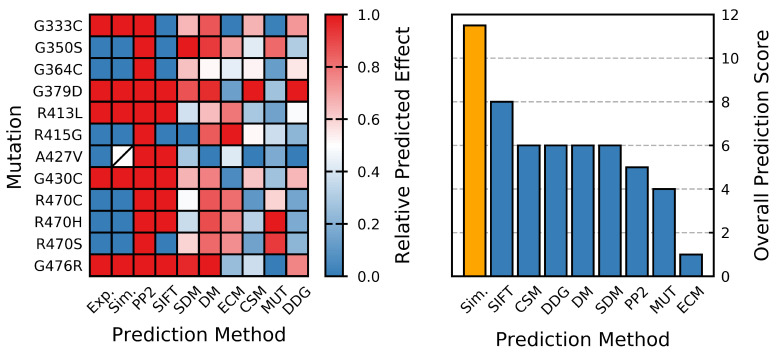
Performance of methods for predicting protein stability. Relative predicted effect (destabilizing: red, stabilizing: blue) is based on the normalized value for each method (most destabilizing mutation predicted by a given method is assigned a score of 1, while the most stabilizing is assigned a score of 0). In some cases, the assignment is strictly binary. Overall predictive score is based on the number of “correct” predictions (number of predicted mutation effects that agree with previous experimental findings). Abbreviations are used for some of the in silico methods, and these are indicated as follows: Exp.: In vitro effect [70,71,72], Sim.: predicted effect from the MD analysis performed herein, PP2: PolyPhen-2 [21], SIFT [22], SDM [27], DM: DynaMut [46], ECM: EnCoM [32], CSM: mCSM [37], MUT: I-Mutant 3.0 [23,24] and DDG: DeepDDG [33].

The dynamic structural analysis clearly indicates that different mutants have different effects on protein structure, and MD simulations can produce ensembles describing behavior that is in close agreement with the reported effects [70,71,72]; specifically, that the G333C, G379D, R413L, G430C and G476R mutants are destabilizing, while the G350S, G364C, R415G, A427V, R470C, R470H and R470S mutants are neutral/stabilizing. Our results demonstrate that MD can perform very well in predicting the effects of mutations on the Kelch domain of KEAP1 (Figure 12) and can provide insights that are often inaccessible to static in silico methods.

For five of the mutations, G333C, G379D, R413L, G430C and G476R, the structural analysis points to effects that are destabilizing: significant decreases in the anti-parallel β-strand content, large shifts in the dihedral sampling space and loss of hydrogen bonds all indicate mutagenic effects that are destabilizing. Not all the destabilizing mutations appear to have the same effects on the backbone dihedral angles and hydrogen bonding, however. Mutations of the structural glycines, G333C, G379D and G430C, all resulted in significant shifts in the backbone dihedrals, while hydrogen bonding was not severely affected. Conversely, the R413L and G476R mutations resulted in more constrained shifts in the backbone dihedrals. The local hydrogen bonding network, however, both directly and indirectly, was significantly disrupted.

For three of the mutations, G350S, G364C and R415G, the structural analysis is similarly clear and points to effects that are neutral/stabilizing. High S${}_{a\beta }$, limited shifts in the backbone dihedrals, and limited changes in the hydrogen bonding networks all suggest mutagenic effects that are neutral or even improve stability.

For all but one of the nine mutations, A427V, the observed dynamic behavior is distinctly either destabilizing (G333C, G379D, G430C, R413L and G476R) or neutral/stabilizing (G350S, G364C, R415G, A427V and R470C/H/S), and agrees with published findings (stabilizing: G350S, G364C, R415G and R470C; destabilizing: G333C, G379D, G430C, R413L and G476R) [70,71,72]. A427V showed decreases in S${}_{a\beta }$; however, we observed no significant shift in the backbone dihedrals under mutation. Furthermore, favorable increases in the hydrogen bonding, and only a limited increase in the RMSD, all suggest this mutant does not have a significant effect on stability; however, given the S${}_{a\beta }$, we classify it as inconclusive.

We note that in using the findings [72] that involved the attempted purification of the wild type and the nine mutant Kelch proteins, we assume that the insoluble mutants were destabilizing and those that could be purified were neutral/stabilizing. There is an inherent limitation in such an assumption as protein solubility is not entirely dependent on protein stability. The inability to work with some of these protein mutants experimentally, as was the case in this previous work [72], underscores one of the shortcomings of experimental methods and highlights how MD can be used to extract additional information from systems that are difficult, if not impossible to analyze at the bench.

As a protein stability prediction method, our plain-MD approach showed an above-average accuracy when compared to other in silico methods. Concluding that those mutations that result in significant decreases in S${}_{a\beta }$ are destabilizing, and that those that result in minimal changes or increases are neutral/stabilizing, gives this method an overall predictive accuracy of 89% assuming the correctness of previous studies [70,71,72] and that the R470C and associated variants (R470H and R470S) are neutral/stabilizing [70,71]. This compares well to eight alternative in silico methods (Figure 12, Table 1). Given the performance of the S${}_{a\beta }$ for classifying the effect of amino acid substitutions on Kelch stability, future studies looking at this domain may benefit from utilizing S${}_{a\beta }$ as a collective variable in a metadynamics-based approach [97].

### 4.2. β-Blades I, II and III Are Less Stable Than Blades IV, V and VI

The blade-wise analysis of the wild-type structure clearly indicates lower S${}_{a\beta }$ values and higher fluctuations in β-blades I, II and III, and higher S${}_{a\beta }$ values and lower fluctuations in β-blades IV, V and VI (Figure 4). Furthermore, mutations G333C, G379D, R413L and G430C, all located in blades I-III, show highly destabilizing effects that are almost entirely localized to these same blades (Figure 3). Interestingly, according to the COSMIC database, the missense mutation count of the six blades of Kelch is 71, 64, 79, 62 (+24 ANCHOR mutant site), 45 and 53, respectively; that is, the total number of mutations in the first three blades is 214, and the total in the final three is 160 (+24). Given this, it is tempting to hypothesize that the cancerous mutations in Kelch preferentially cluster in structurally significant regions in order to destabilize the domain and weaken NRF2 binding.

### 4.3. ANCHOR Mutant

The group of mutants referred to as “additionally NRF2-complexed hypomorphs” (ANCHOR) are thought to confer an increased binding affinity to KEAP1 for NRF2 [70,71]. One of these mutants, R470C, sits distant (Figure 1) from the NRF2 binding site of the Kelch domain in KEAP1 and is believed to allosterically increase affinity via backbone perturbations, resulting in a conformational change in the binding pocket [71]. This is somewhat paradoxical as R470C is the most frequently listed missense mutation in KEAP1 in the COSMIC database [69].

Dynamic analysis of the residues in the Kelch domain predicted to hydrogen bond with NRF2, reveals that R470C, R470H and R470S have relatively unchanged fluctuations at these sites compared to the wild type, with R470C and R470H tending to show the smallest fluctuations overall (Figure 11). With respect to global dynamics, the R470C and R470H mutations both resulted in increases in S${}_{a\beta }$ and decreases in RMSD compared to the wild type. Whether these minor fluctuations induced by R470C result in increased binding affinity, as previously postulated [71], cannot be concluded. However, in agreement with these previous results, changes to the binding pocket residues were observed. Although differences in the mutagenic effects are evident, why it is that R470C occurs twice as frequently as R470H and eight times as frequently as R470S in the COSMIC database is not entirely explained by these dynamics data.

It is a documented phenomenon that, on a proteome level in cancer, net gain of cysteine, histidine and tryptophan and a net loss of arginine occur [98]. These mutations may confer a unique beneficial phenotype to cancer. One possibility is that the mutation to cysteine provides another site for post-translational modification (PTM). C470 is highly solvent accessible with a relative solvent accessibility of 0.58 and has a predicted pKa of 7.73, making it a possible nucleophile and target for electrophilic covalent modification [99]. Several other cysteines, C368 (pKa: 6.93), C434 (pKa: 6.63), C489 (pKa: 6.99) and C583 (pKa: 7.29), are known to be modified in the Kelch domain and have predicted pKa values similar to that at C470 and all of these are below the pKa of a normal cysteine thiol (∼9.5) [91,100].

Modification by sulforaphane has been previously shown to result in NRF2 activation. While C151 is the predominant sulforaphane sensor in KEAP1, C489 and C368, both in the Kelch domain, have been shown to readily form adducts with sulforaphane [101,102]. Additionally, previous computational modeling revealed that modification of C434 and C368 by glutathione resulted in structural changes in Kelch that would impair KEAP1/NRF2 binding [103], and modification of C434 by 8-nitro-cGMP has been shown to activate NRF2 [104]. We do note that Eggler et al. previously found that modification of the cysteines of KEAP1 is insufficient to significantly disrupt binding to NRF2, and that disruption of the KEAP1-CUL3 interaction is the probable mode of action [105]. In any case, a modification of R470C may still partially explain its observed frequency in the cancer database. Given the nature of the KEAP1-NRF2 pathway, increased dissociation would result in increased nuclear accumulation and the upregulation of key genes that are believed to confer a chemoprotective phenotype to cancer cells [68].

Additional MD simulations using a PTM-parameterized forcefield [78,106,107] and a mutant structure with an explicitly modified cysteine is an approach that may provide additional insight into the possible mutagenic effect of R470C. While our findings do not rule out the possibility of a direct allosteric effect of the R470C mutant on the binding site, an absence of significant structural dynamics at the binding site points to a less trivial explanation.

## 5. Conclusions

Our work provides additional insight into the structural dynamics of mutants that could not be analyzed experimentally, painting a more complete picture of their mutagenic effects [72]. These findings also appear to point to a preferential targeting of protein stability in cancer mutants, which to our knowledge is being reported for the first time. In addition, our analysis points to S${}_{a\beta }$ (Equation (1)) being a superior metric compared to RMSD for characterizing the effects of the mutations on the Kelch propeller. While this cannot be employed for all systems, when applicable, the use of S${}_{a\beta }$ or the associated parallel β-strand (S${}_{p\beta }$) and α-helical (S${}_{\alpha }$) order parameters [86] would provide insights beyond the standard RMSD, RMSF and radius of gyration metrics. Furthermore, our results reveal that the R470C ANCHOR mutant induces only minor structural changes in the binding site of Kelch relative to the wild type, suggesting that a direct allosteric effect of the mutant may not fully explain its potential mode of action and frequent occurrence in the cancer database.

## Figures and Tables

**Table 1:** Performance of methods for predicting mutagenic effect. PP2, SIFT and PMC use dimensionless values, while the remaining methods use ΔΔG (kcal/mol). Abbreviations are used for some of the in silico methods and these are indicated as follows: Exp.: In vitro effect [70,71,72], Sim.: predicted effect from the MD analysis performed herein, PP2: PolyPhen-2 [21], SIFT [22], PMC: PoPMuSiC [29], SDM [27], DM: DynaMut [46], ECM: EnCoM [32], CSM: mCSM [37], MUT: I-Mutant 3.0 [23,24] and DDG: DeepDDG [33]. Exp. and Sim. categories use the following abbreviations: destabilizing (D), neutral/stabilizing (S/N) or inconclusive (I).

Mutant	Exp.	Sim.	PP2	SIFT	PMC	SDM	DM	ECM	mCSM	MUT	DGG
G333C	D	D	1.00	0.05	/	−1.45	−0.76	1.25	−1.92	−0.63	−1.98
G350S	S/N	S/N	0.99	0.22	/	−3.40	−1.00	0.19	−1.13	−1.15	−0.69
G364C	S/N	S/N	1.00	0.03	/	−1.37	0.81	0.35	−1.48	−0.70	−1.46
G379D	D	D	1.00	0.00	/	−2.72	−1.15	0.98	−2.99	−0.79	−2.83
R413L	D	D	1.00	0.00	−0.58	0.04	0.24	−0.38	−0.68	−0.71	−1.23
R415G	S/N	S/N	0.95	0.43	/	2.18	−0.70	−0.80	−1.42	−0.86	−0.40
A427V	S/N	I	1.00	0.00	−0.59	0.57	3.16	0.40	0.22	−0.73	0.26
G430C	D	D	1.00	0.00	/	−1.52	−0.33	1.14	−1.80	−0.79	−1.77
R470C	S/N	S/N	0.88	0.01	/	−0.51	−0.75	−0.42	−0.13	−1.00	−0.22
R470H	S/N	S/N	1.00	0.03	/	−0.14	−0.84	−0.31	−0.82	−1.26	−0.28
R470S	S/N	S/N	0.99	0.08	/	−1.12	−0.57	−0.27	−0.23	−1.21	−0.42
G476R	D	D	1.00	0.00	/	−3.19	−1.36	0.75	−0.99	−0.62	−2.11

## Data Availability

Simulation data are available from the authors upon reasonable request.

## Supplementary Materials

The following are available online at https://www.mdpi.com/article/10.3390/ijms22105408/s1, Figure S1: Wild type time-dependent structural properties, Figure S2: Mutant time-dependent RMSD, Figure S3: Mutant time-dependent moving RMSD (mRMSD), Figure S4: Mutant time-depedent radius of gyration (Rg), Figure S5: Residue-wise difference contact map, Table S1: Mutagenic effect on the hydrogen bonding network of the Kelch domain, Table S2: Effect of G476R mutant on the hydrogen bonding of R507.
