# Supplementary material for: KEAP1 Cancer Mutants: A Large-Scale Molecular Dynamics Study of Protein Stability
Source: Int J Mol Sci. 2021 May 20;22(10):5408. doi: 10.3390/ijms22105408 (PMC8161161; doi:10.3390/ijms22105408)
Supplement: Supplementary file 1 [file ijms-22-05408-s001.zip › ijms-1175351-supplementary.pdf]

Supporting information: KEAP1 cancer mutants: A large-scale molecular dynamics study of protein stability

Carter J. Wilson,[†,‡] Megan Chang,[†] Mikko Karttunen,[*,¶,‡,§] and Wing-Yiu Choy[*,†]

†*Department of Biochemistry, The University of Western Ontario, 1151 Richmond Street, Canada, N6A 5C1*

‡*Department of Applied Mathematics, The University of Western Ontario, 1151 Richmond Street, Canada, N6A 5B7*

¶*Department of Chemistry, The University of Western Ontario, 1151 Richmond Street, Canada, N6A 3K7*

§*Centre for Advanced Materials and Biomaterials Research, The University of Western Ontario, 1151 Richmond Street, London, Ontario, Canada, N6A 5B7*

E-mail: mkarttu@uwo.ca; jchoy4@uwo.ca

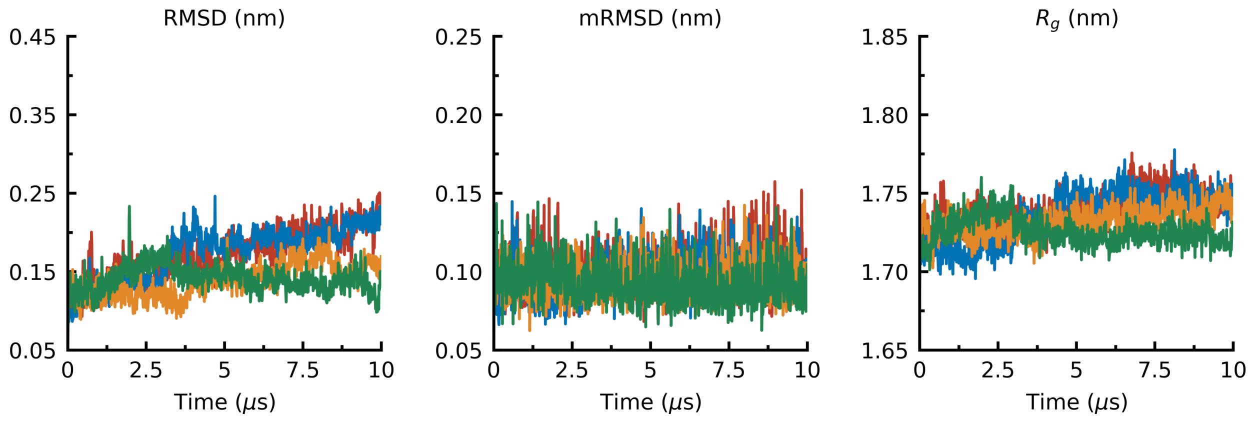

Figure S1: Wild type time-dependent structural properties. The backbone RMSD, moving backbone RMSD (mRMSD) (computed between the current frame and the frame 1 ns previous), and radius of gyration (R_g), are shown as a function of simulation time. Colors are used to distinguish between individual runs.

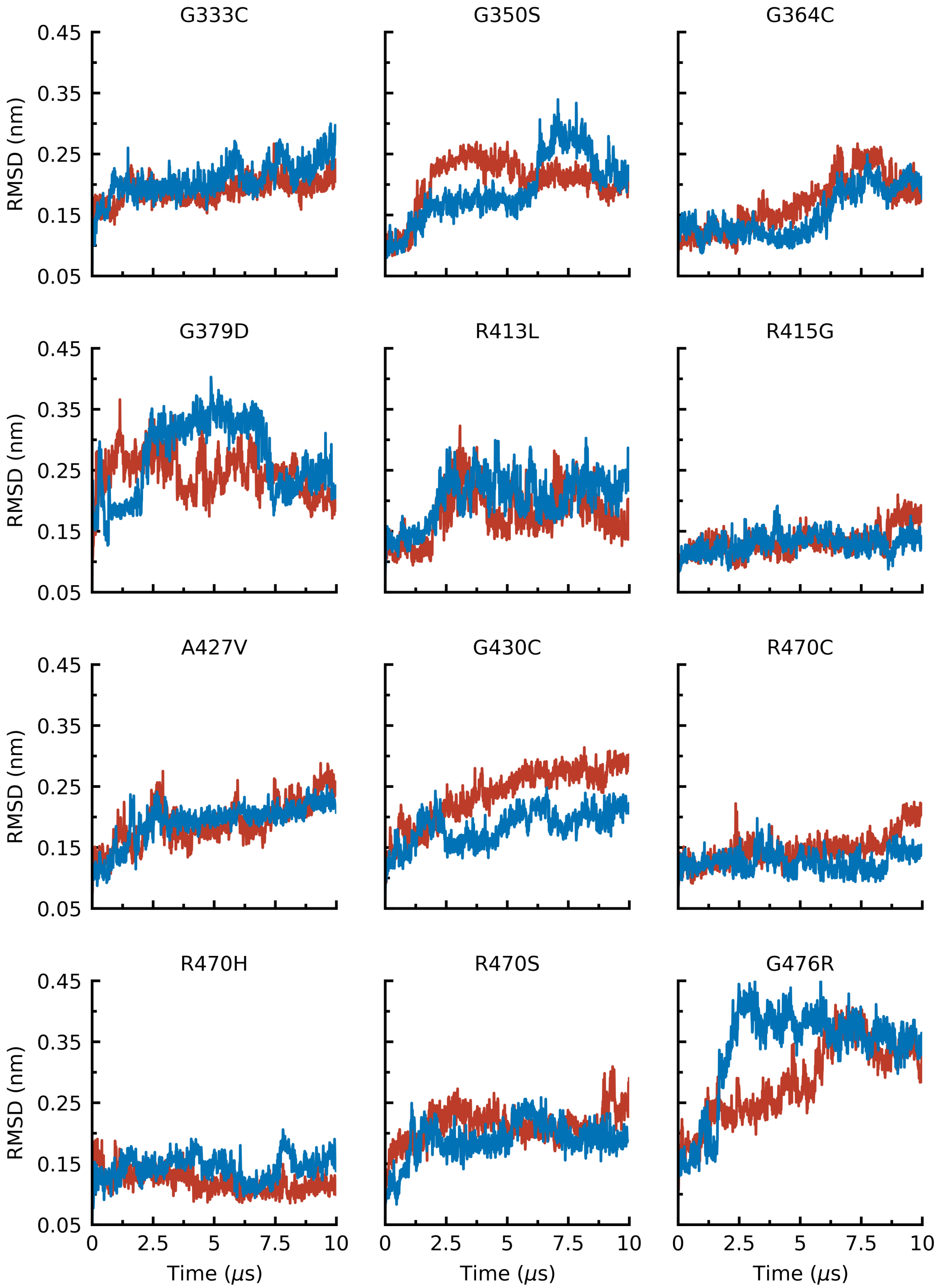

Figure S2: Mutant time-dependent RMSD. The backbone RMSD is shown as a function of simulation time. Colors are used to distinguish between individual runs.

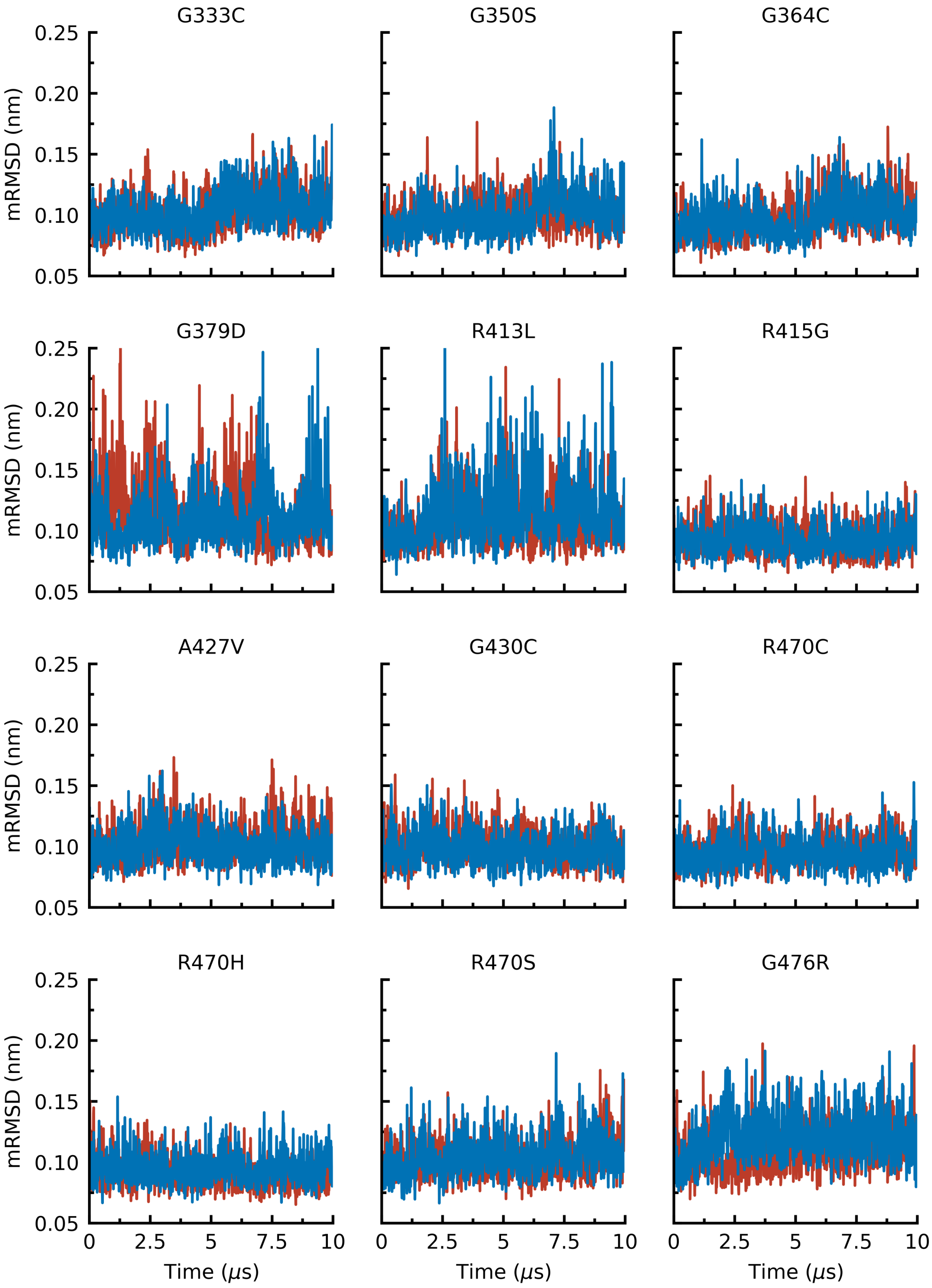

Figure S3: Mutant time-dependent moving RMSD (mRMSD). The backbone mRMSD (computed between the current frame and the frame 1 ns previous) is shown as a function of simulation time. Colors are used to distinguish between individual runs.

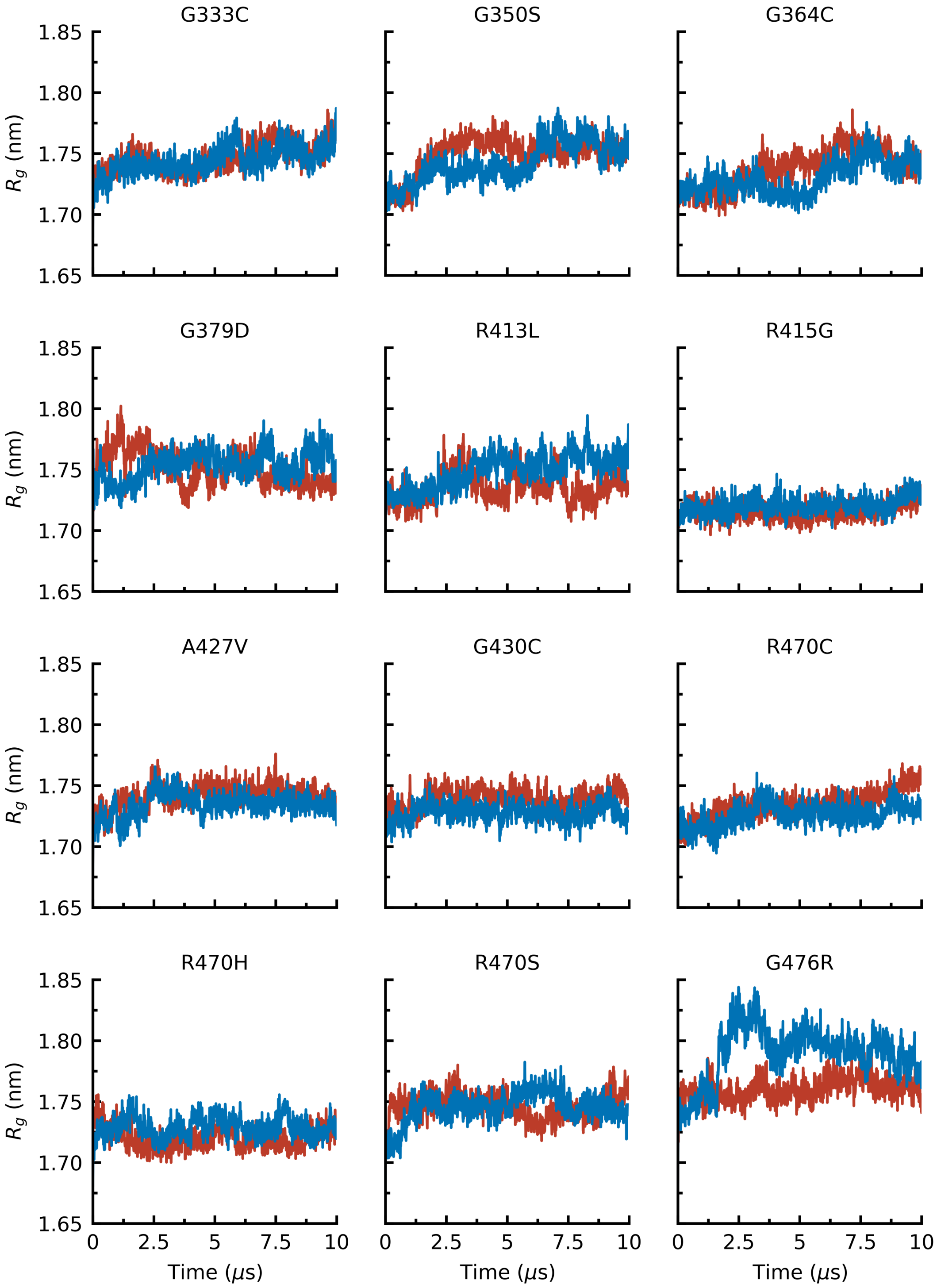

Figure S4: Mutant time-depedent radius of gyration (R_g). The R_g is shown as a function of simulation time. Colors are used to distinguish between individual runs.

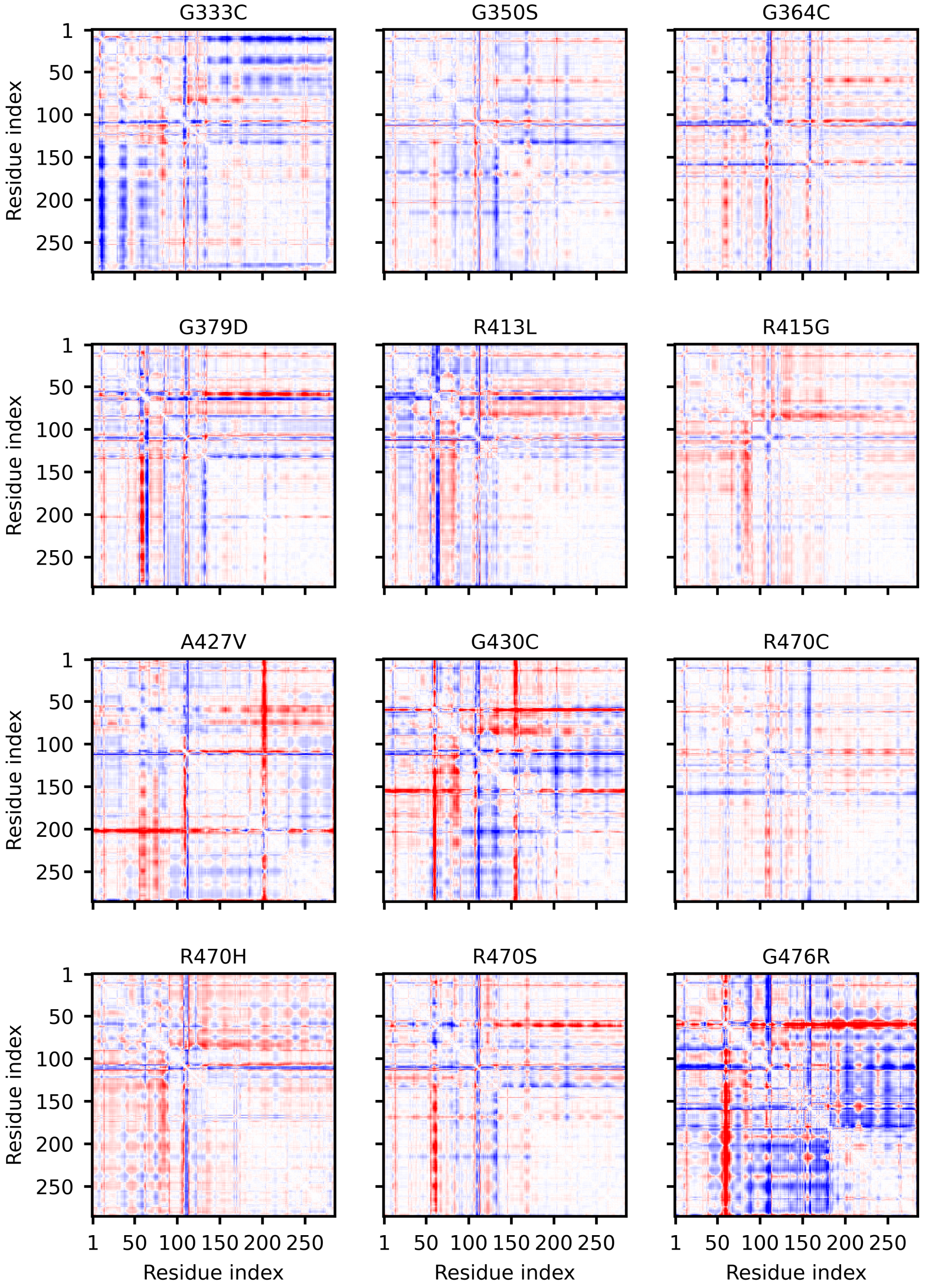

Figure S5: Residue-wise difference contact map. The average distance, over the final 100 ns, was computed between the center of masses of each residue. This was subtracted from the average distances in wild type. A blue through red colormap was used, where blue indicates longer distances relative to wildtype and red indicates shorter distances relative to wildtype.

Table S1: **Mutagenic effect on the hydrogen bonding network of the Kelch domain.** Lifetimes of hydrogen bonds in wild type and the mutant structures. Only hydrogen bonds that existed for $\geq 20\%$ of the sampled trajectory are considered. The change under mutation, rounded to the nearest whole number, is also recorded. Sampling was performed over the final $5\,\mu$s of trajectory.

Donor	Acceptor	Wild type	Mutant	Change
G333 (N)	R362 (O)	54.5	0.0	55 decrease
L339 (N)	G333 (O)	52.7	38.7	24 decrease
G364 (N)	G603 (O)	45.5	39.1	6 decrease
G364 (N)	S363 (OG)	23.6	30.8	7 increase
G379 (N)	R413 (O)	44.1	22.1	22 decrease
G379 (N)	S363 (O)	0.0	22.1	22 increase
S390 (N)	G379 (O)	56.7	0.0	57 decrease
R415 (N)	G379 (OD1)	0.0	22.6	23 increase
R413 (N)	S390 (O)	40.2	0.0	40 decrease
R413 (NH1)	G430 (O)	21.7	0.0	22 decrease
R413 (NH2)	E441 (OE1)	22.4	0.0	22 decrease
R413 (NH2)	E441 (OE2)	20.8	0.0	21 decrease
R415 (N)	N414 (OD1)	0.0	39.6	40 increase
G462 (N)	R415 (O)	23.8	32.9	9 increase
A427 (N)	E441 (O)	76.1	79.7	4 increase
E441 (N)	A427 (O)	26.7	44.0	17 increase
G430 (N)	R460 (O)	29.2	0.0	29 decrease
H437 (N)	G430 (O)	39.0	0.0	39 decrease
N414 (N)	G430 (O)	0.0	31.3	31 increase
R470 (NE)	D422 (OD1)	22.0	0.0	22 decrease
R460 (NH1)	G476 (O)	42.0	0.0	42 decrease
G476 (N)	S486 (O)	0.0	28.9	29 decrease

Table S2: **Effect of G476R mutant on the hydrogen bonding of R507.** Lifetimes of hydrogen bonds in wild type and the G476R mutant. Only hydrogen bonds that existed for $\geq$ 10% of the sampled trajectory are considered. The change under mutation, rounded to the nearest whole number, is also recorded. Sampling was performed over the final 5 μs of trajectory.

Donor	Acceptor	Wild type	Mutant	Change
R507 (N)	L484 (O)	34.0	0.0	34 decrease
R507 (NH1)	G523 (O)	63.7	0.0	64 decrease
R507 (NH1)	S533 (O)	61.1	0.0	61 decrease
R507 (NH1)	I506 (O)	0.0	20.5	21 increase
R507 (NH2)	S533 (OG)	61.1	0.0	61 decrease
R507 (NH2)	E535 (OE1)	13.6	19.9	3 increase
R507 (NH2)	E535 (OE2)	60.5	19.6	41 decrease
R507 (NH2)	T505 (OG1)	22.8	0.0	23 decrease
R507 (NE)	E535 (OE1)	0.0	16.7	17 increase
R507 (NE)	E535 (OE2)	0.0	14.7	15 increase
G477 (N)	R507 (O)	58.9	0.0	59 decrease
